# Everybody's Page

**Published:** 1888-07-21

**Authors:** 


### EVERYBODY'S PAGE.

CERTAIN diseases, such as measles, scarlet fever, &c., are likely to be accompanied with or followed by eye affections.

YOU do not want much force to stop a bleeding vessel in the human body, provided that you place your finger in the proper position. The strength of the little finger in the puniest living child is enough to control almost any common bleeding vessel, were the finger applied at the required point.

REGULAR work for the brain, even in tender years, is good, provided that there are periods of repose, and that the organism, as a whole, is healthy. If the work be injudiciously arranged, or the system be feeble, headaches and languor, loss of appetite and arrest of nutrition herald a general breakdown.

WITH primitive races an illness, like any other interruption of the regularity of natural phenomena is, as a rule, attributed to the operations of some malevolent power. The Maories of New Zealand, for instance, have, or had, a special devil named Tonga, whose malign influence was held responsible for any headache that might occur.

THE nerves which lie embedded in the lining membrane of the nose warn our senses of the presence of hurtful gases. But they do more than that, for if some particles of an irritating nature gets into the nose, these nerves set in motion a very singular mechanism, by which the intrusive particles are expelled by sneezing, so that they may not find their way downwards into the air-passages and do harm.

EVERY one knows what it is to have particles of food pass into tbe windpipe, or "go down the wrong way" as it is called. This is very bad for the air passages and lungs, and Nature steps in to remedy it. Small dust particles are greatly got rid of by means of the little hair-like cilia which are always sweeping upwards. Small particles of food are, however, far too large to be got rid of in this way, and therefore another means is provided. That means is cough.

THE large towns of England are very deadly to child-life. Within a year of birth they destroy on an average 185 of every thousand born. If we take individual towns, the sacrifice is still more dreadful. For example, in Liverpool it is 219 out of every thousand born, and in Leicester it sometimes rises as high as 245; that is to say, one out of every four of the unhappy babies of Leicester is buried within twelve months of its birth.

IF we look more closely at a town as compared with a rural population, we discover various characteristic features which it is of interest and importance for us to note. There is always in towns a much greater proportion of adults of both sexes, persons at the prime of life. The marriageable females are greatly in excess of the males of the same age. As a natural consequence, early marriages are much more frequent in the towns than in the rural districts, the birth rate is higher, and the proportion of children under five years of age is in excess. But between five and fifteen years of age the proportion is reversed. Though the town population is more productive, it is less conservative of child-life than the rural population. Another equally sinister characteristic of a town population is that, while above forty-five in country districts, the proportion of persons still living in the married state is maintained, even to extreme old age; in the towns it falls off in comparison, and is replaced by a large excess of widows and widowers.

As the result of numerous experiments, we have learned that the simplest way of purifying air is to saturate it with a mixture of steam and heat. Sulphurous acid is often used to purify rooms, but it is practically useless. Chlorine is more useful, but it does not penetrate sufficiently to kill all dangerous organisms. If, however, we saturate the atmosphere of any room with a mixture of steam and hot air, almost every organism is destroyed.

WHAT a beautiful thing is charity, especially intelligent charity! It is stated that a Spanish magistrate, shocked and exasperated by repeated food adulterations, has issued a proclamation, aflame with righteous wrath, ordaining that "all wines, groceries, and provisions which, upon analysis, are proved to be injurious to health, will be confiscated forthwith and distributed to the different charitable institutions." Does the worthy magistrate think that what is injurious to health can be helpful in disease, or is he moved by an economic desire to relieve charity, public and private, of the burden of supporting the sick? The conundrum is an interesting one.

WHEN children have had a fall in the streets, or fall off chairs, etc., the most common way of rendering assistance, and the most thoughtless way, is to place them on their feet at once, as if the mere remaining on the floor after a fall was all the discomfort which required attention. The small weight of the child, the short height the body has to fall, and the extreme elasticity of the tissues, all combine to render ordinary falls in children comparatively harmless; but should there happen to be serious injury, placing them on their feet is the best method of adding very considerably to that injury.

DENTINE, which forms the body of the tooth, is composed of an infinite number of excessively minute tubes embedded in a matrix of calcareous or lime salts, so that it consists of something like bone. These tubes are arranged so as to lie with one end opening towards the pulp at the centre of the tooth, the other terminating at its external surface, each of them measuring in the diameter of its bore about one ten-thousandth of an inch. Their use seems to be the maintenance of the life of the tooth by securing its nutrition from the central cavity found running along each tooth, which cavity contains the blood-vessels and nerves necessary for keeping it alive.

IT is well known in many districts that if plants are burned potash, one of the constituents of soap, can be obtained from the ashes. So common is this practice in certain countries that an amusing anecdote is told of an accident which happened when an ancient tomb was opened recently in Rome. One of the workmen finding some ashes in the tomb, collected them and took them home to his wife, so that she might treat them with water and cleanse the family linen; but it came out afterwards that instead of the ashes being from wood they were the cremated remains of the Emperor Galba, who had been interred there 1,800 years ago. It is to be feared the good housewife did not get much cleansing potash out of these ashes.

THE attempt to limit the growth of Paris by prohibiting the erection of new buildings beyond certain bounds was begun in 1549, and renewed from time to time down to 1672, when Louis XIV. justified his edict by the following reasons: "(1) That by enlarging the city, the air would be rendered unwholesome. (2) That cleaning the streets would prove a great additional labour. (3) That adding to the number of inhabitants would raise the price of provisions, of labour, and of manufactures. (4) That ground would be covered with buildings instead of corn, which might hazard a scarcity. (5) That the country would be depopulated by the desire that people have to resort to the capital. (6) That the difficulty of governing such numbers would be an encouragement to robbery and murder."

